# Maximizing Identification Precision of Hymenoptera and Brachycera (Diptera) With a Non‐Destructive DNA Metabarcoding Approach

**DOI:** 10.1002/ece3.70770

**Published:** 2025-01-23

**Authors:** Isabel C. Kilian, Ameli Kirse, Ralph S. Peters, Sarah J. Bourlat, Vera G. Fonseca, Wolfgang J. Wägele, Andrée Hamm, Ximo Mengual

**Affiliations:** ^1^ Museum Koenig Bonn Leibniz Institute for the Analysis of Biodiversity Change Bonn Germany; ^2^ Agroecology and Organic Farming Group, Institute of Crop Science and Resource Conservation (INRES), Faculty of Agriculture University of Bonn Bonn Germany; ^3^ Centre for Environment, Fisheries and Aquaculture Science (Cefas) Weymouth Dorset UK

**Keywords:** agriculture, bulk samples, clustering algorithms, COI, Malaise trap, molecular units, Syrphidae

## Abstract

In recent years, DNA metabarcoding has been used for a more efficient assessment of bulk samples. However, there remains a paucity of studies examining potential disparities in species identification methodologies. Here, we explore the outcomes of diverse clustering and filtering techniques on data from a non‐destructive metabarcoding approach, compared to species‐level morphological identification of Brachycera (Diptera) and Hymenoptera of two bulk samples collected with Malaise traps. The study evaluated four distinct approaches, namely clustering to Amplicon Sequence Variants (ASVs) or ASVs clustered to Operational Taxonomic Units (OTUs) coupled with subsequent filtering using the LULU algorithm at 84% and 96% minimum match. In total, 114 species of Brachycera (35 families) and 85 species of Hymenoptera (27 families) were identified morphologically. Depending on the selected approach, DNA metabarcoding results strongly varied in terms of detected molecular units blasted to brachyceran and hymenopteran species. For Brachycera, ASVs clustered into OTUs followed by LULU using a 96% minimum match (OTU96) inferred the number of molecular units closest to the number of morphologically identified species. Using Syrphidae as an exemplary family, we found an overlap ranging from 9% to 81% between the morphological identification and the different clustering and filtering approaches, OTU96 being also here the closest one. For Hymenoptera, while OTU96 also yielded the highest number of molecular units, it was still considerably low compared to the number of morphologically identified species. Our results show that metabarcoding methodology needs to be significantly improved to be applied to Hymenoptera. Conversely, for Brachycera, we acknowledge the promise of employing a non‐destructive metabarcoding approach, incorporating ASV clustering into OTUs and filtering with LULU, to derive dependable species lists. Such lists hold significant potential for applications in biomonitoring, conservation efforts, and other related fields.

## Graphical abstract

DNA metabarcoding is utilized for efficient bulk sample assessment, yet limited research addresses disparities in species identification methods. We compared diverse clustering and filtering techniques in non‐destructive metabarcoding approach to morphological identification of Brachycera and Hymenoptera. Results varied notably in detected molecular units for brachyceran and hymenopteran species, emphasizing the need for improved metabarcoding methodologies for Hymenoptera and the potential of non‐destructive approaches for Brachycera in biomonitoring and conservation applications.

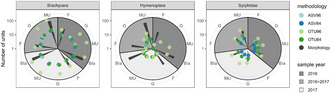

## 1 Introduction

In view of a worldwide insect decline (Van Klink et al. 2020), large‐scale biomonitoring initiatives on the basis of standardized protocols are more important than ever. Malaise trapping is a well‐established method to collect flying insects, and they have been extensively utilized in various local (Geiger, Moriniere, et al. 2016; Hallmann et al. 2017) and global biodiversity assessment initiates (e.g., Global Malaise Program; https://biodiversitygenomics.net/projects/gmp/). Malaise traps are non‐attractant, static interception traps, which consist essentially of an open‐fronted tent with a trapping device attached to the inner highest corner of the tent (Henderson and Southwood 2016; Muirhead‐Thompson 1991; Townes 1962). Diptera and Hymenoptera are usually the most specimen and species‐rich insect taxa found in Malaise trap catches (Matthews and Matthews 1971; Skvarla et al. 2021). Despite their significance, many contemporary studies utilizing Malaise trap samples often lack detailed species‐level information (Hallmann et al. 2017).

DNA metabarcoding is frequently utilized for assessing arthropod diversity from bulk samples (deWaard et al. 2019; Huang et al. 2022; Wägele et al. 2022). It is capable of identifying thousands of specimens in parallel by analyzing with high‐throughput sequencing (HTS) (Taberlet et al. 2012). A common practice in DNA metabarcoding to yield high DNA quantities involves homogenizing the entire sample (Beng et al. 2016; Gibson et al. 2015). However, by homogenizing the sample, undetected and rare species are irreversibly destroyed, thereby hindering a subsequent morphological identification (Carew, Coleman, and Hoffmann 2018; Kirse et al. 2023). Our knowledge of flying insect diversity in Central Europe and beyond, particularly in Diptera and Hymenoptera, remains limited, highlighting the importance of preserving morphological vouchers (see, e.g., Hausmann, Segerer, et al. 2020). Therefore, new developments in DNA extraction protocols for metabarcoding are shifting toward non‐destructive extraction methods, such as DNA extraction directly from incubated lysis buffers (Batovska et al. 2021; Carew, Coleman, and Hoffmann 2018; Kirse et al. 2021, 2023; Morinière et al. 2016; Zizka et al. 2018) as well as from preservative ethanol (Kirse et al. 2023; Zenker, Specht, and Fonseca 2020), instead of using homogenized tissue samples. To infer putative species from metabarcoding raw data of Malaise trap samples (or other bulk samples), sequences can be clustered into either Operational Taxonomic Units (OTUs, also known as Molecular Operational Units or MOTUs) or Amplicon Sequence Variants (ASVs). OTUs are clustered sequences based on a fixed similarity threshold (Kopylova et al. 2016; Westcott and Schloss 2015), while ASVs are zero radius OTUs, encompassing only sequences that exhibit zero genetic distance from any other sequence in the dataset (Porter and Hajibabaei 2018). Although different terminologies may be used in practice for the Amplicon Sequence Variants, such as ASV, zero radius OTUs (zOTU), and ESV (Exact Sequence Variant), they essentially refer to similar methods (Antich et al. 2021; Nearing et al. 2018). For simplicity, we will use the term ASV hereafter.

OTUs come with two major limitations. First, while OTUs are often used as a proxy for species (Porter and Hajibabaei 2018), if closely related species exhibit only limited variation in the barcode region and the clustering threshold is not appropriately chosen, it can artificially reduce the number of species detected. Second, OTUs are only valid within the dataset they have been created in, meaning comparison across datasets is only feasible when the data are being combined and reanalyzed. In contrast, ASV tables can be compared across datasets, because the ASV approach clusters sequences without a threshold and infers groups already based on a single‐nucleotide difference (Callahan, McMurdie, and Holmes 2017). Several studies have demonstrated that ASVs often represent the true ecological situation and diversity patterns as well or even more accurately than OTUs (Callahan et al. 2016; Joos et al. 2020; Porter and Hajibabaei 2018, 2020). However, taxa exhibiting high levels of intraspecific variation are prone to be represented by multiple ASVs, thereby artificially inflating the number of detected putative species (Callahan, McMurdie, and Holmes 2017). In addition, both OTUs and ASVs can generate artificial clusters due to sequencing errors, leading to discrepancies in the number of actual species present in the sample (Koeppel and Wu 2013; Schloss and Westcott 2011). To mitigate the occurrence of erroneous molecular units, algorithms have been developed for post‐clustering curation of resulting OTU and ASV tables such as AMPtk (Palmer et al. 2018), dbOTU3 (Olesen, Duvallet, and Alm 2017), or LULU (Frøslev et al. 2017). LULU assesses the pattern of sequences present in lower counts, often arising from sequencing or polymerase chain reaction (PCR) artifacts, to curate the list and filter out misleading ASVs or OTUs. A major advantage of LULU is its independence from a reference database, as it integrates read abundance with the degree of minimum match (sequence similarity). Minimum_match is one of the user‐selected parameters representing the minimum threshold difference between sequences from the cluster for considering any OTU as an error. After analyzing the initial OTU or ASV table with the LULU algorithm, a new OTU table is constructed. Some studies have already shown that abundance filtering alone may lead to over‐filtering, resulting in an underestimation of overall OTU diversity, since OTUs with low read counts could be erroneously filtered out (Callahan et al. 2016; Frøslev et al. 2017).

Comparative studies between species identified through clustering DNA metabarcoding data and those identified morphologically are still relatively scarce (Beentjes et al. 2019; Huo et al. 2020; Topstad et al. 2021), particularly within the realm of insects (Kirse et al. 2023; Mata et al. 2021; Remmel et al. 2024; Zenker et al. 2016). These studies also provided diverse results, ranging from similar taxonomic composition (Remmel et al. 2024) to highly different, caused by a higher (Topstad et al. 2021) or lower (Beentjes et al. 2019) number of species identified with DNA metabarcoding. Moreover, such comparisons often rely on mock communities to facilitate analysis. While mock communities serve as a robust tool for systematically comparing different methodologies (Iwaszkiewicz‐Eggebrecht et al. 2023; Nielsen et al. 2019), studies involving bulk samples (e.g., Malaise trap samples) may yield different outcomes due to the high complexity of samples (Marquina et al. 2019). To our knowledge, no study has yet evaluated the outcomes of different clustering and filtering approaches based on ASVs or OTUs, and filtered with LULU at different minimum_match settings, against the results from morphological identification of species across diverse flying insect taxa. In this study, we compared the overlap between species identification with a non‐destructive DNA metabarcoding approach coupled with four different clustering and filtering approaches with the morphological identification of adult Brachycera and Hymenoptera from bulk samples collected with Malaise traps.

## 2 Material and Methods

### 2.1 Study Area and Sample Collection

**FIGURE 1:**
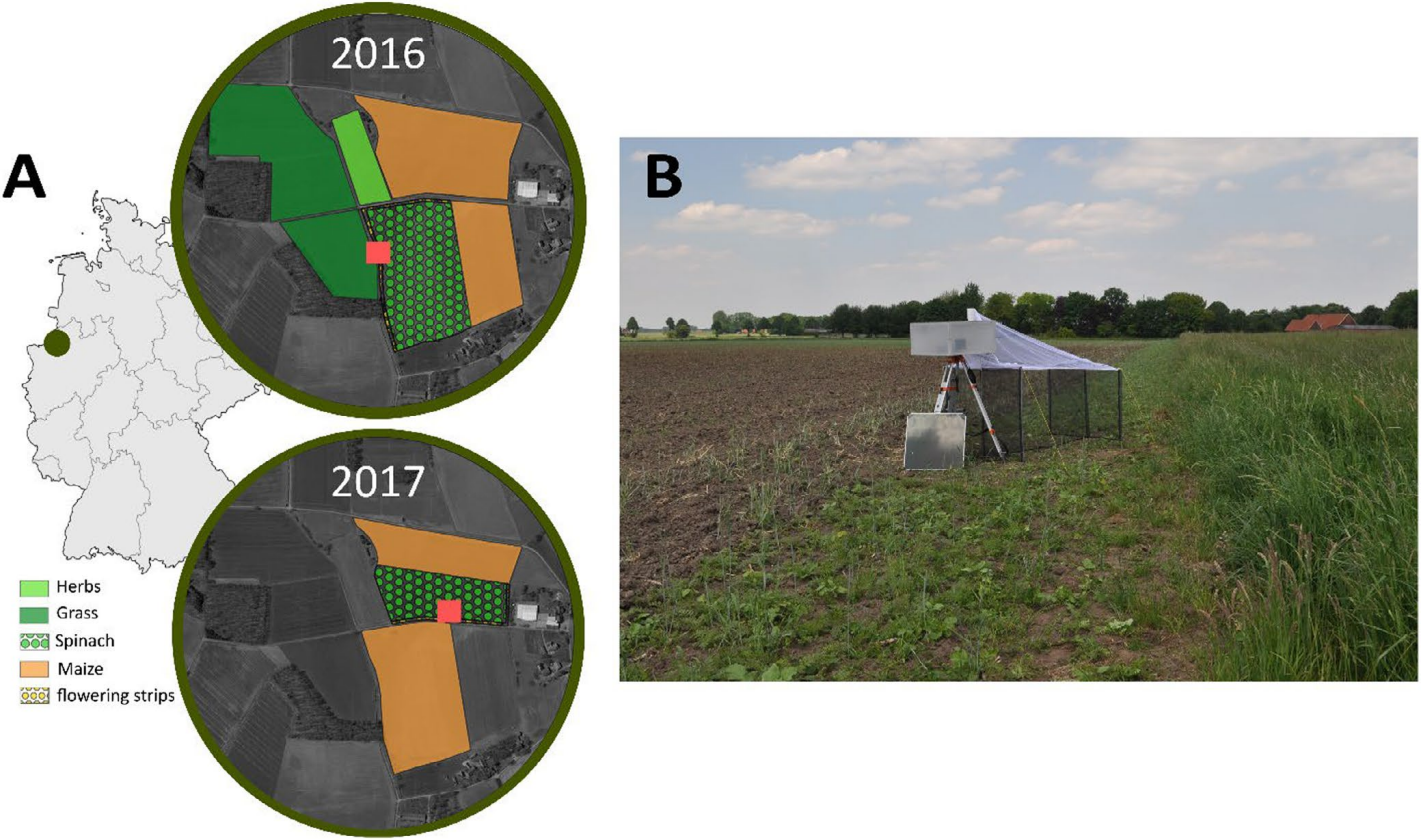
(A) Map with the location of the (B) Multi‐sampler attached to a Malaise trap (in red) located in 2016 and 2017 in spinach fields with flowering strips.

Study sites were located in the area of Borken, north‐western Germany (51.807765 N/6.832369 E in 2016; 51.810295 N/6.830871 E in 2017). The area is dominated by agricultural fields with maize, spinach, and other non‐flowering plants (Meyhöfer, Klug, and Poehling 2008; Figure 1A). For the collection of the Brachycera and Hymenoptera specimens, an automated multi‐sampler unit attached to a commercial Townes‐style Malaise trap (Kirse et al. 2024; Wägele et al. 2022) was set up on spinach fields with flowering strips in 2016 and 2017 (Figure 1B). Spinach ( *Spinacia oleracea* L.) is the most important field‐grown vegetable in the area of Borken, with an annual harvest of around 34,000 tons. This constitutes approximately half of the total harvested in Germany, with the majority being processed into frozen food (FAOSTAT 2022; Frerichs and Daum 2021). Since spinach is usually harvested before the flowering period, and thus not a primary attractant for flowering‐visiting insects, there is a local interest in increasing biodiversity in spinach‐dominated areas through the implementation of flower strips. Malaise traps were positioned directly at the border between the spinach field and the flowering strips with 1000 mL collection bottles filled with 96% ethanol. Following retrieval from the field, the samples were kept in 96% ethanol and stored at room temperature. The study presented here is based on two bulk samples, collected during two periods: from August 24 to 31, 2016, and from July 4 to 11, 2017, respectively.

### 2.2 Morphological Identification

Adult specimens assigned to Hymenoptera and Brachycera from both bulk samples were counted and identified to morphospecies level based on morphological characters (Table S1) and to species level in the case of the family Syrphidae (Diptera, Brachycera). The highly diverse superfamily Ichneumonoidea (Hymenoptera) were excluded from the analysis due to a lack of specific expertise within our team, and the identification of morphospecies based solely on external features can be inadequate (Horstmann 2002; Veijalainen et al. 2011). The sorted samples are deposited as vouchers at the Museum Koenig Bonn (Leibniz Institute for the Analysis of Biodiversity Change).

### 2.3 DNA Extraction and Analysis

DNA extraction was carried out following a modified protocol from Aljanabi and Martinez (1997) (Vesterinen et al. 2016). Initially, the ethanol in the bulk sample was decanted from the bottles using the MICROFILV Filter (White Gridded 0.45‐μm‐Dia 47 mm and 100 mL Funnel Sterilized) equipped with a 0.45 μm filter membrane to retain small individuals and body parts. The remaining insects were dried for 10 min. Due to the high biomass of both bulk samples and to ensure thorough contact of all the specimens with the extraction buffer, we divided each sample into four equal subsamples (Figure S1). Subsequently, each subsample was mixed with 50 mL of extraction buffer (0.4 M NaCl, 10 mM Tris–HCl pH 8.0, 2 mM EDTA pH 8.0, and 2% sodium dodecyl sulfate (SDS) of the final concentration). Extraction negative controls just with the extraction buffer were added. Additionally, 400 μg Proteinase K per mL of lysis buffer was added to each subsample. Subsamples were then incubated for digestion overnight at 52°C on an orbital shaker set at 200 rpm. After digestion, the lysis solution from each subsample was evenly divided into three 50 mL falcon tubes, resulting in three replicates per subsample (24 samples in total, two samples × four subsamples × three replicates; Figure S1). The lysate was filtered using MICROFILV Filter (White Gridded 0.45 μm‐Dia 47 mm and 100 mL Funnel Sterilized) equipped with a 0.45 μm filter membrane to filter out the insects. After this second filtering step, we once again pooled the 24 samples into six extraction triplicates (three for each sample; Figure S1), which were processed separately throughout the remaining protocol and kept separate until the bioinformatic analysis. In the subsequent step, each tube received an additional 1.12‐fold amount of lysis solution containing 6 M NaCl. The tubes were then vortexed for 30 s before being centrifuged for 30 min at 3556 g. The supernatant was carefully transferred to new tubes, to which an equal amount of isopropanol was added. The solution was gently mixed by inverting the tubes upside down a few times before placing them at −20°C for 1 h. Following this, the tubes were centrifuged at 4°C and 3556 g for 60 min. The solution was carefully decanted and 2 mL of −20°C 70% EtOH was added to the remaining pellet. The tubes were centrifuged at 4°C and 3556 g for 15 min. Subsequently, the supernatant was discarded and tubes with the remaining pellet were left to dry overnight at room temperature. Afterward, pellets in each tube were dissolved in 1 mL of sterile H_2_O at room temperature for 4 h. DNA extracts were quantified using the Quantus Fluorometer (Promega) and stored at −20°C until further processing.

### 2.4 Library Preparation Strategy

Library preparation was conducted following a two‐step PCR approach (Bourlat et al. 2016; Fonseca and Lallias 2016). The first PCR (amplicon PCR, PCR1) was carried out using amplicon‐specific primers with Illumina adapter overhangs and the second (index PCR, PCR2) allowed the incorporation of Illumina index adapters (Bourlat et al. 2016). The 313‐bp‐long mitochondrial cytochrome *c* oxidase subunit I (COI) region of interest was amplified using the forward primer mlCOIintF (5′‐GGWACWGGWTGAACWGTWTAYCCYCC‐3′) (Leray et al. 2013) and the reverse primer jgHCO2198 (5′‐TAAACTTCAGGGTGACCAAAAAATCA‐3′) (Leray et al. 2013), yielding a suitable fragment size for both performing with higher success rates than other primer sets (e.g., LCO1490/HCO2198) in NGS applications (Leray et al. 2013).

Approximately, 10 ng of template DNA was used for all PCR reactions. Additionally, two PCR‐negative controls (with no template DNA) were added. For the amplicon PCR, the mastermix used in this study consisted of 7.5 μL Q5 Hot Start High‐Fidelity 2× Master Mix (New England BioLabs), 5 μL Sigma H_2_O, 0.5 μL of forward Primer, 0.5 μL of reverse primer, 0.5 μL Bovine Serum Albumin (Thermoscientific), and 1 μL of template DNA, making up a total volume of 15 μL. The amplicon PCR was initialized by denaturation of 2 min at 98°C, which was followed by 20 cycles with 40 s at 98°C, 40 s at 45°C, 30 s at 72°C, and a final extension of 3 min at 72°C. PCR1 products were purified with HT ExoSAP‐ITTM (Applied Biosystems) by adding 4 μL of HT ExoSAP‐ITTM to each sample. Following the manufacturer's protocol, samples were incubated for 15 min at 37°C, followed by 15 min at 80°C before being cooled down for 5 min at 4°C. For the index PCR, 8 μL of purified PCR1 products was used. The purified PCR products were therefore split into two PCR tubes. Each tube contained 12.5 μL Q5 Hot Start High‐Fidelity 2× MasterMix (New England BioLabs), 3 μL Sigma H_2_O, 1.2 μL of forward primer, 1.2 μL of reverse primer, and 8 μL purified PCR1 product. Again, an initial denaturation step of 2 min at 98°C was applied, followed by 20 cycles with 40 s at 98°C, 30 s at 55°C, 30 s at 72°C, and a final extension of 3 min at 72°C. PCR2 products were visualized by gel electrophoresis and purified using the QIAquick Gel Extraction Kit (Qiagen), according to the manufacturer's instructions. All final purified amplicons (PCR2) were quantified using the Quantus Fluorometer (Promega) and diluted to the same concentration (3 ng/μL) before pooling. The resulting purified amplicon pools were sequenced on an Illumina Miseq (2 × 300 bp) sequencing platform at Liverpool University's Centre for Genomic Research (UCGR, Liverpool). The raw data have been deposited at the Genbank SRA archive under accession number PRJNA1105927.

### 2.5 High‐Throughput Sequencing Data Analysis

An initial quality check was carried out at the UCGR. The raw fastq files were trimmed for the presence of Illumina adapter sequences using Cutadapt (v.1.2.1) (Martin 2011). Additionally, sequences were trimmed using Sickle (v.1.200) with a minimum window quality score of 20. Reads shorter than 20 bp were removed after trimming. Additionally, demultiplexing was carried out by the sequencing company.

**FIGURE 2:**
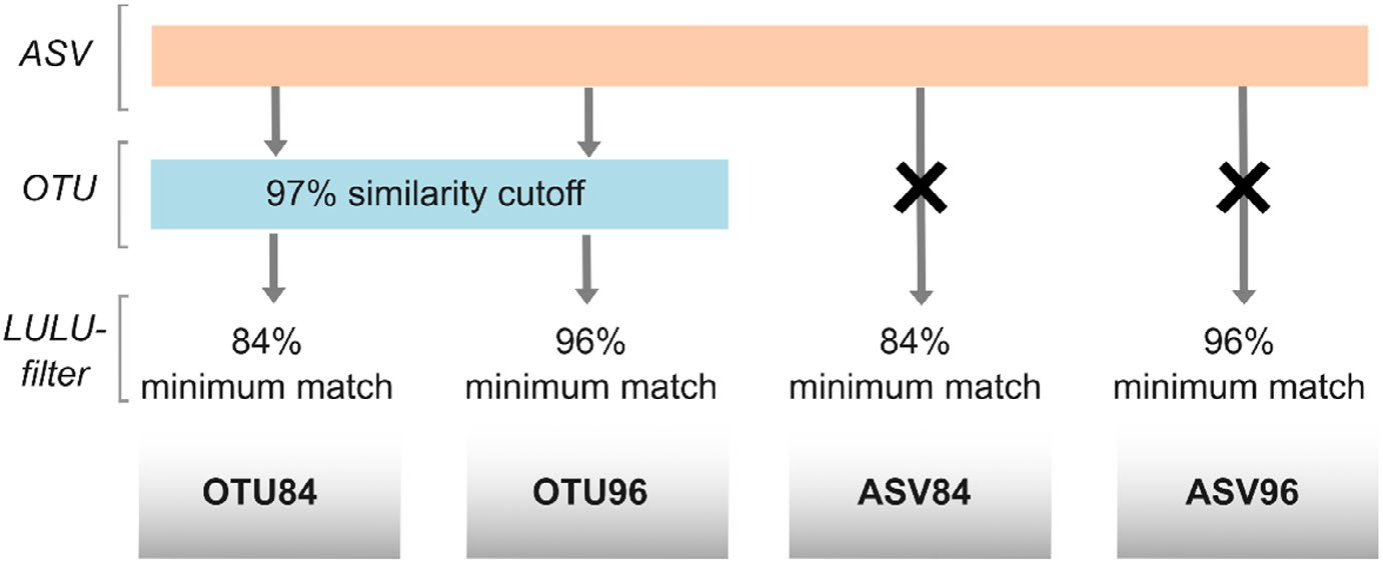
Schematic differences between the four different clustering approaches: ASVs were either clustered in OTUs at 97% cutoff and LULU‐filtered using default settings at 84% minimum match (OTU84) or using a 96% minimum match (OTU96), or ASVs were just directly LULU‐filtered using the default settings at 84% minimum match (ASV84) or (4) at 96% cutoff (ASV96).

The raw fastq files were trimmed for the presence of COI primers using Cutadapt (v.1.18) using the following settings: maximum error rate (−e): 0.1, minimum overlap (−O): 20, and minimum sequence length (−m): 50. Only sequences containing both forward and reverse primers were kept for further analyses. Subsequently, filtered and trimmed raw reads without the primer pairs were uploaded to QIIME2 (v.2022.2) (Bolyen et al. 2019). In an initial filtering step, all forward reads were truncated to 269 bp and reverse reads to 274 bp. Further analysis steps including paired‐read merging, quality filtering, and denoising were conducted with the implemented DADA2 version (Callahan et al. 2016). We then started four different clustering and filtering approaches starting with either ASVs or ASVs clustered to OTUs at a 97% similarity cutoff. Initially, we used a BLAST search of ASV and OTU representative sequences, respectively, against each other using BLASTN (v.2.9.0) with the following settings: “query coverage high‐scoring sequence pair percent” (−qcov_hsp_perc) was set to 80 and minimum percent identity (−perc_identity) was set to 84 (default setting). To filter for erroneous sequences, the post‐clustering filter algorithm LULU (v.0.1.0) (Frøslev et al. 2017) was applied either directly to the ASVs or OTUs dataset with the (1) minimum match set at 84% (from here on referred to as ASV84 or OTU84) or (2) minimum match at 96% (ASV96 or OTU96) (Figure 2). We used the default value of the minimum_match parameter in LULU (84%) and selected another higher value (96%)—“a higher value is recommended for markers with little variation and/or few expected PCR and sequencing errors” (Frøslev et al. 2017). Then, for each dataset, the number of sequences found in the negative PCR controls was subtracted from the according OTUs or ASVs. For the data analysis, we aggregated the total number of reads per molecular unit from each extraction triplicate per sample (Figure S1) and converted it into a binary present–absence dataset. Finally, the taxonomic assignment was carried out against the BOLD database (https://www.boldsystems.org) using BOLDigger (access date: 06.03.2023; Buchner and Leese 2020), including early‐release and private records. The output list was filtered using the JAMP‐Pipeline method implemented in BOLDIGGER (Buchner and Leese 2020). In detail, assignments to different taxonomic levels were conducted according to the following similarity thresholds: 98% species, 95% genus, 90% family, 85% order, and < 85% class (Elbrecht et al. 2017). For instance, for a 96% hit, the species‐level assignment will be discarded and genus‐level information will be used as the lowest taxonomic level.

We manually checked the output list for possible synonyms (same species with different scientific names) to facilitate comparisons between the different methods. Moreover, we computed Shannon's index (*H*′) (*vegan* package; Dixon 2003) and Pielou's measure of species evenness (*E*) (*chemodiv* package; Petrén, Köllner, and Junker 2023) based on the morphological dataset to gain a better understanding of both bulk samples. To compare morphology with the different clustering and filtering approaches, we focused on two different species diversity components: (1) species richness, defined as the number of identified molecular units, morphospecies, or species (in the case of Syrphidae); and (2) species composition (applied only for Syrphidae), representing the species community of Syrphidae identified using the different methodologies. For the analysis of the Syrphidae species composition, we used the Jaccard dissimilarity index (*J*) (*vegan* package, Oksanen et al. 2016). All the species diversity and composition analyses were performed in R (v.1.4) with the *tidyverse* package (Wickham et al. 2019), while the heatmaps were additionally generated using the *cowplot* package (Wilke, Wickham, and Wilke 2019) combining data from both samples.

## 3 Results

### 3.1 Morphological Identification

**FIGURE 3:**
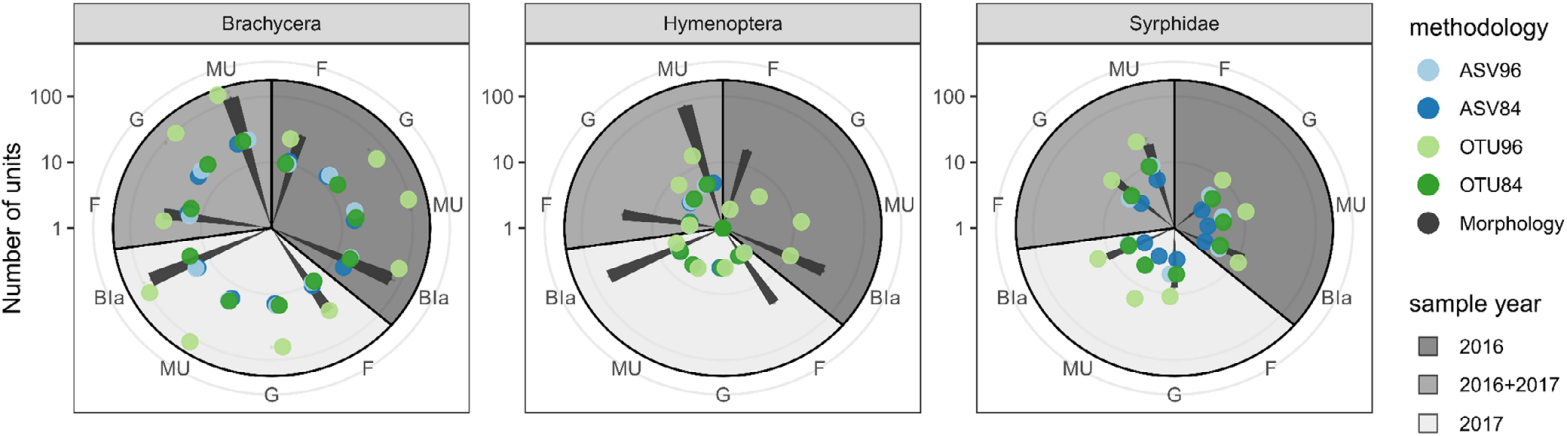
Comparison of number of units at different taxonomic levels of Brachycera, Hymenoptera, and Syrphidae detected with morphological identification (bars) and DNA metabarcoding with four different clustering approaches (colored dots) across two samples of 2016 and 2017, and combined results. ASVs were either directly LULU‐filtered at 96% minimum match (ASV96) or using the standard setting at 84% minimum match (ASV84), or ASVS were first clustered to OTUs (at 97% similarity cutoff) and afterward LULU‐filtered at 96% minimum match (OTU96) or using the standard setting at 84% minimum match (OTU84). Bla = blasted molecular units, morphospecies or species; F = family; G = genera; MU = molecular units; Logarithmic scale.

In the 2016 sample, we identified a total of 839 brachycerans and 533 hymenopterans (*H*′ = 3.23, *E* = 0.694) sorted into 71 and 36 morphospecies, respectively, belonging to 29 Brachycera and 17 Hymenoptera families. In the 2017 sample, we identified a total of 1189 Brachycera and 813 Hymenoptera specimens (*H*′ = 3.39, *E* = 0.693) sorted into 75 and 59 morphospecies, respectively, belonging to 31 Brachycera and 22 Hymenoptera families. Combining both sampling years, we identified a total of 114 species of Brachycera (35 families) and 85 species of Hymenoptera (27 families) (Figure 3, Table S2).

In 2016, Drosphilidae was the most abundant family in Brachycera (225 specimens), followed by Syrphidae (144 specimens), while Tenthredinidae (56 specimens) and Proctotrupidae (14 specimens) were the most abundant among Hymenoptera. In 2017, Anthomyiidae was the most abundant family of Brachycera (493 specimens) followed by Hybotidae (107 specimens), while Apidae (187 specimens, of which 110 specimens were identified as *Bombus lucorum* (Linnaeus, 1761)) and Tenthredinidae (62 specimens) had the highest number of individuals for Hymenoptera. Regarding morphospecies richness, Syrphidae (12 species) and Tenthredinidae (11 morphospecies) were the most diverse families in 2016, whereas Pteromalidae (12 morphospecies), Tachinidae (11 morphospecies), and Syrphidae (11 species) exhibited the highest morphospecies richness in 2017.

Concerning the species diversity of Syrphidae, the most abundant species in both years was *Melanostoma mellinum* (Linnaeus, 1758): 109 specimens in 2016 (75.7% of the total number of hoverflies), while 45 specimens in 2017 (43.7% of the total number of hoverflies). Additionally, four syrphid species in 2016 and five syrphid species in 2017 were singletons, that is, each species was represented by just one specimen (Table S3).

### 3.2 DNA Metabarcoding Assessment

DNA metabarcoding directly from the lysis buffer with all four analysis approaches recovered between 180,010 and 189,562 reads from the bulk sample of 2016. Specifically, OTU84 generated 180,010 reads, ASV84 produced 181,983 reads, OTU96 resulted in 189,562, and ASV96 yielded 180,010 reads. Similarly, for the bulk sample of 2017, the DNA metabarcoding approach retrieved between 244,564 and 244,501 reads. OTU84 and ASV84 both generated 244,586 reads, OTU96 produced 244,501 reads, and ASV96 resulted in 244,586 reads.

In 2016, we detected between 15 and 97 Brachyceran molecular units depending on clustering and filtering method, corresponding to 15–80 species across 11–24 families. For the Hymenoptera, we detected 1–13 Hymenoptera molecular units matching 1–11 species across one to two families (Figure 3, Table S2). Similarly, in 2017, we detected between 16 and 119 Brachyceran molecular units matching 16–96 species across 11–30 families. Hymenoptera exhibited five molecular units corresponding to five different taxa across five families using all four different clustering methods. Overall, OTU96 detected the highest number of reads and species in both orders (Figure 3, Table S2).

Within the family Syrphidae, 11 species were identified with OTU96, five species with OTU84 or ASV96, and three species with ASV84 in 2016. In total, three species were identified using all four clustering and filtering approaches. In 2017, the situation was similar, with 15 species identified with OTU96, five species with OTU84 or ASV96, and three species with ASV84. Again, three species were identified using all four clustering and filtering approaches. Additionally, one hoverfly molecular unit detected with OTU84 in 2017 could not be matched to any taxon in the reference database (Table S3).

### 3.3 Comparison of Morphological Identification and Metabarcoding

**FIGURE 4:**
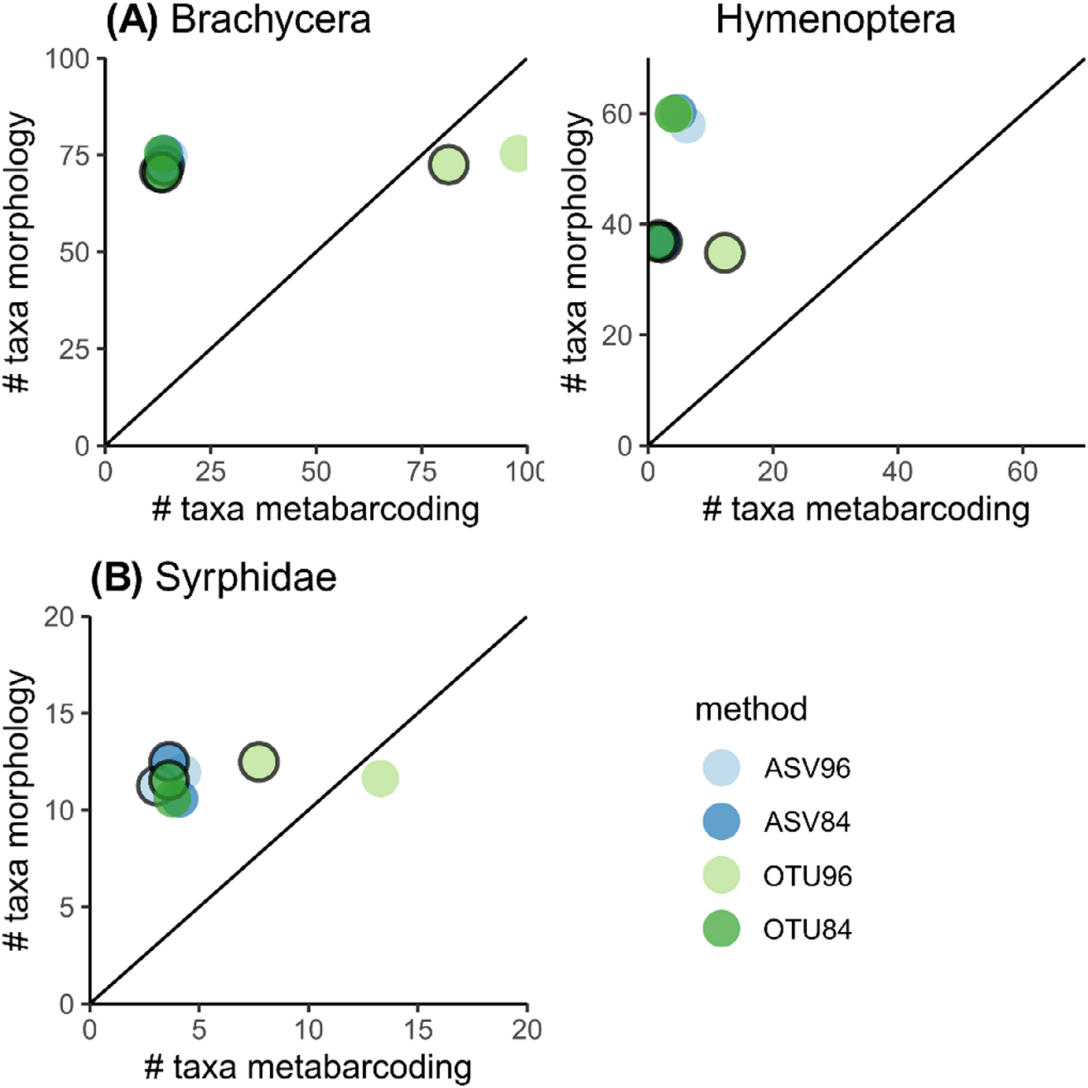
Comparison between the number of (A) hymenopteran and brachyceran taxa and (B) Syrphidae species detected with morphological identification and DNA metabarcoding with four different clustering approaches across two samples of 2016 and 2017. ASVs were either directly LULU‐filtered at 96% minimum match (ASV96) or using the standard setting at 84% minimum match (ASV84), or ASVS were first clustered to OTUs (at 97% similarity cutoff) and afterward LULU‐filtered at 96% minimum match (OTU96) or using the standard setting at 84% minimum match (OTU84). The solid line represents a 1:1 relationship. Points with black borders represent the sample of 2016, and points without border represent the sample of 2017.

**FIGURE 5:**
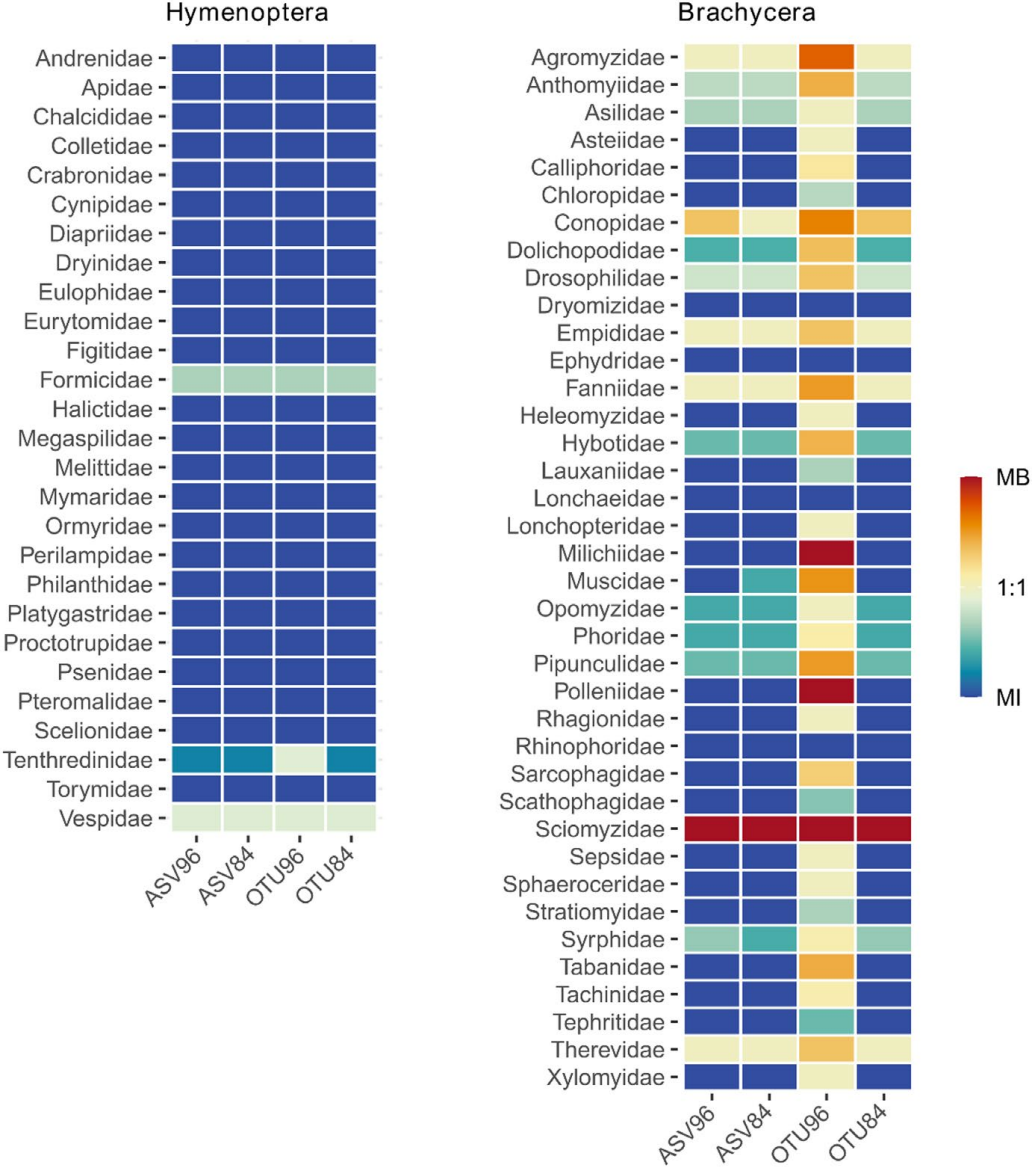
Comparison of the ratio between four different bioinformatic approaches (ASV filtered with LULU using a 96% [ASV96] or 84% minimum match [default setting; ASV84], ASV clustered to OTUS at 97% similarity cutoff and LULU curated at 96% [OTU96] or at 84% minimum match [OTU84]) and the morphological identification of Hymenoptera and Brachycera. Morphological and metabarcoding identification could have either identified the same number of taxa (1:1) or inclined to identify more taxa with metabarcoding (MB, red) or via morphological identification (MI, blue).

**FIGURE 6:**
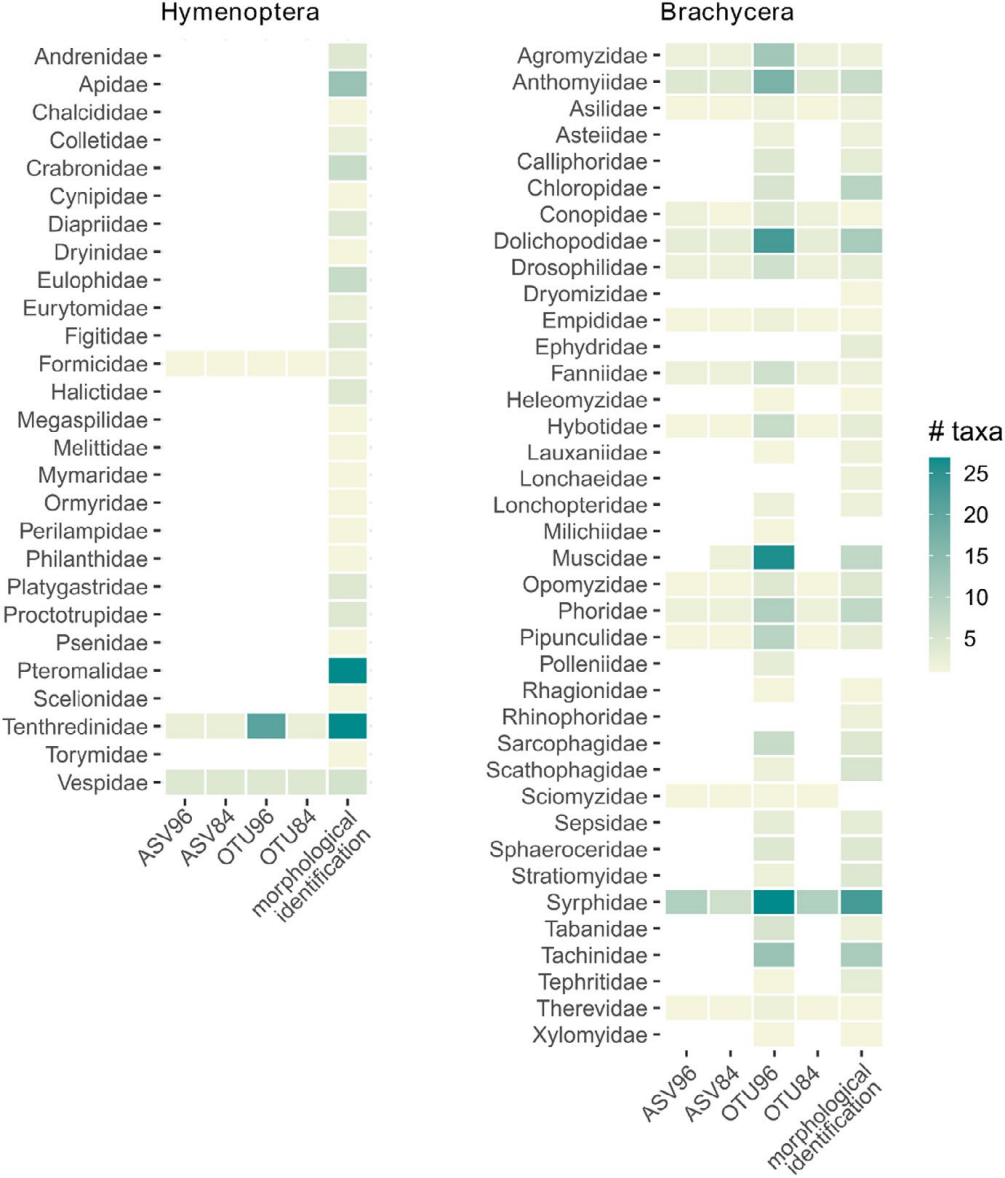
Heatmap comparing the number of hymenopteran and brachyceran species identified morphologically and via DNA metabarcoding either by LULU‐curated ASV using a 96% (ASV96) or 84% minimum match (default setting; ASV84), or ASV clustered to OTUS at 97% similarity cutoff and LULU curated at 96% (OTU96) or at 84% minimum match (OTU84), of two samples collected in 2016 and 2017.

DNA metabarcoding revealed different numbers of putative species (molecular units) in Hymenoptera and Brachycera depending on which of the four different clustering and filtering approaches was used (Figure 4). For Brachycera across both sampling years, ASV84, ASV96, and OTU84 underestimated the number of the species identified using morphology (in Figure 4, point above the 1:1 crossline), while OTU96 was the closest though notably overestimating the number of species (in Figure 4, points below the 1:1 crossline). Among the brachyceran families identified by adult morphology, nine were not found by any of the clustering and filtering approaches, namely Dryomizidae, Ephydridae, Heleomyzidae, Lonchaeidae, Rhinophoridae, Stratiomyidae, Tephritidae, Therevidae, and Xylomyidae (Figures 5 and 6). Contrarily, three families were not identified using morphology in the sample, but with DNA metabarcoding only: Sciomyzidae was detected through all four clustering and filtering approaches, while Milichiidae and Polleniidae were identified specifically with OTU96. The pattern of underestimation of brachyceran species richness, evident across all clustering and filtering approaches, except for OTU96, is also apparent for Syrphidae. Although a slight underestimation of species number persists in the sample of 2016, OTU96 exhibited the closest species number to those identified using morphological characters (i.e., the closest to the 1:1 crossline) (Figure 4).

For Hymenoptera, all clustering and filtering approaches substantially underestimated the species number identified by morphological characters (in Figure 4, point above the 1:1 crossline), with OTU96 providing the closest estimation of species numbers in both samples (Figure 4). Among the taxa identified by morphology, *Athalia rosae* (Linnaeus, 1758) (Tenthredinidae) and *Lasius niger* (Linnaeus, 1758) (Formicidae) were also identified using DNA metabarcoding (Figures 5 and 6). While Vespidae was also identified using both DNA metabarcoding and morphology, the two species of this family retrieved by DNA metabarcoding were not found morphologically. Moreover, 25 additional families identified morphologically were not determined by any of the clustering and filtering approaches (Figures 5 and 6).

In terms of similarities in syrphid species composition, OTU96 showed the highest similarity with morphology for both samples (*J* = 0.67 in 2016 and *J* = 0.4 in 2017). The same highest Jaccard value was found for the 2017 sample with ASV84 and ASV96 (*J* = 0.4) (Table 1). *Melanostoma mellinum* , as the most abundant species identified with morphology in both years, was detected with all four clustering and filtering approaches (Table S3). However, none of the syrphid singletons from 2016 was found using DNA metabarcoding. In contrast, in the 2017 sample, four out of the six singletons were identified with OTU84. Lastly, nine syrphid species (seven in 2016 and two in 2017) identified using morphology were not detected by DNA metabarcoding (Table S3). Contrarily, different metabarcoding approaches detected 13 syrphid species (six in 2016 and seven in 2017) that were not found in the morphology study, being OTU96 (in the 2016 sample) and OTU84 (in the 2017 sample) the metabarcoding approaches with the highest number of syrphid species detected that were not present in the morphological study (six each) (Table S3).

**TABLE 1:** Jaccard dissimilarity index (*J*) of Syrphidae between the different samples based on morphological data (morpho) and DNA metabarcoding with four different clustering and filtering approaches, based on a binary dataset. The closer the value is to 0, the higher the similarity is between the methods. OTU84 = ASVs clustered in OTUs at 97% similarity cutoff and LULU‐filtered using the standard settings at 84% minimum match, ASV84 = ASVs and LULU‐filtered using standard settings at 84% minimum match, ASV96 = ASV and LULU‐filtered using a 96% minimum match, OTU96 = ASV clustered to OTUs at 97% similarity cutoff and LULU‐filtered using a 96% minimum match.

Comparison	2016 (*J*)	2017 (*J*)
OTU84:morpho	0.69	0.5
ASV84:morpho	1	0.4
ASV96:morpho	1	0.4
OTU96:morpho	0.67	0.4

## 4 Discussion

To our knowledge, this study represents the first comparative analysis of species‐level diversity in bulk samples (focusing specifically on Brachycera and Hymenoptera) comparing the diversity assessments from traditional adult morphology with those obtained through a non‐destructive DNA metabarcoding approach, placing particular emphasis on testing various clustering and filtering methods, combining ASVs, OTUs, and LULU curation. Among the four different clustering and filtering approaches tested here, OTU96 emerges as the method that better reflects species richness identified by morphological characters and closely approximates species composition for Brachycera, specifically in Syrphidae. This alignment between DNA metabarcoding and morphological identification for Brachycera mirrors the findings from similar studies on arthropod diversity in Malaise trap samples (Remmel et al. 2024) and freshwater invertebrates (Beentjes et al. 2019; Cahill et al. 2018).

Previous research has extensively examined the advantages and disadvantages of using OTU and ASV clustering, primarily in the context of microbiome assessments (Barnes et al. 2020; Chiarello et al. 2022) but also for arthropods (Giebner et al. 2020; Porter and Hajibabaei 2020). Some authors argue that ASVs exhibit a clear superiority over OTUs in their ability to identify a larger number of distinct taxa (Giebner et al. 2020; Porter and Hajibabaei 2020). However, other studies suggest that ASVs may lead to an overestimation of diversity due to high levels of intraspecific diversity of the sampled taxa, often combined with a high degree of artificially introduced sequences (e.g., PCR artifacts) (Andújar et al. 2021; Brandt et al. 2021). In contrast, while OTUs mitigate the impact of sequencing noise, they often achieve this by clustering similar interspecific sequences together into a single OTU, which can artificially lower the assessed diversity and is, therefore, a more conservative approach. For our particular dataset, we advocate for an optimal approach of non‐destructive sample analysis, followed by a clustering intraspecific ASVs into interspecific OTUs for a more accurate identification of Hymenoptera and Brachycera species. This process involves grouping closely related sequences from the same species (intraspecific ASVs) together within larger clusters representing different species (interspecific OTUs). By adopting this method, we aim to accurately represent genetic variation within species while providing a comprehensive understanding of overall diversity.

LULU curation at 96% minimum match, compared to the default setting of 84%, enables the reduction of intraspecific ASVs erroneously considered/assigned to different species, ultimately leading to an estimated species number that aligns more closely with morphological identification. Despite the close resemblance in the estimated number of species between OTU96 and the morphological identification, disparities in species number and composition persist (Figure 5 and Table 1). These disparities can cause a misinterpretation of the health and stability of ecosystems, which is critical to assess conservation strategies (Barsoum et al. 2019). These variations primarily stem from either false positives, where species were detected using DNA metabarcoding but not morphologically, or false negatives, where species were identified morphologically but not detected using DNA metabarcoding.

Potential causes for false positives or negatives include cross‐contamination, shared haplotypes of COI, morphological misidentification, and inaccuracies in reference databases. Cross‐contamination often arises from the analysis of DNA traces, for example, gut content of predatory arthropods (Kirse et al. 2023; Iwaszkiewicz‐Eggebrecht et al. 2023; Lynggaard et al. 2019; Reeves et al. 2018). While analyzing trophic interactions can enhance the analysis, it cannot be quantified until which degree it may lead to an overestimation of species richness and a distortion of species composition compared to morphology. Also, some species (here, i.e., *Melanostoma* species) may share haplotypes between species when analyzing the COI gene fragment (Haarto and Ståhls 2014). While LULU curation partially addresses this issue, it does not account for variations in haplotype proportions (Brandt et al. 2021). Furthermore, morphological identification of highly diverse groups like Diptera can also be difficult (Huang et al. 2022), leading to misidentifications due to the lack of differences in morphological characters (here, i.e., females of certain *Platycheirus* and *Sphaerophoria* species). The presence of cryptic species within species complexes can contribute to these false negatives and/or positives. Lastly, erroneous species identifications can result from inaccuracies in reference databases caused by misleading vouchers. Indeed, conducting a thorough validation of the dataset before performing any diversity analysis as presented by Remmel et al. (2024) by cross‐checking the species list with occurrences in the GBIF (Global Biodiversity Information Facility; Telenius 2011) or GBOL databases (German Barcode of Life; Geiger, Astrin, et al. 2016), as well as a double‐check by taxonomists can mitigate some of the misleading results. However, it depends again directly on the quality of further databases and availability of taxonomists, which can be challenging for many understudied insect taxa. The first and direct consequences of the mismatches, false positives or negatives, are an under‐ or overestimation of diversity. In cases where morphological data are lacking for comparison, this can result in inaccurate biodiversity assessments, leading to ineffective conservation plans (Ficetola, Taberlet, and Coissac 2016).

Overall, no single approach stood out as optimal for analyzing Hymenoptera diversity. The notably low number of Hymenoptera OTUs found cannot be solely attributed to the clustering and filtering approaches, as even the unfiltered ASV dataset already exhibited a surprisingly low number of ASVs assigned to Hymenoptera. This suggests that false negative errors likely occurred during sample processing in the laboratory. There are multiple potential explanations for the absence of many hymenopteran species. First, the lack of larger and more common Hymenoptera species (e.g., *Bombus* species with 190 specimens in the sample of 2017), also evidently from the study by Remmel et al. (2024), could potentially be attributed to a primer bias. Primer bias, which refers to the failure of universal primers to amplify certain taxa, has been documented across various taxa (Clark et al. 2020; Piñol et al. 2015) but is particularly noticeable in Hymenoptera samples (Brandon‐Mong et al. 2015; Elbrecht et al. 2019; Yu et al. 2012). This limitation hampers the comprehensive assessment of Hymenoptera diversity when using one primer alone, as it excludes many important species, including numerous important pollinators (Kilian et al. 2023). Second, biomass bias could have directly influenced the species richness of Hymenoptera detected via DNA metabarcoding. This bias arises because species represented by a limited number of specimens or those that are generally smaller in size may yield lower quantities of DNA (Elbrecht et al. 2019; Erdozain et al. 2019). This could explain why nine brachyceran families represented by a low number of specimens and small in size were not identified at all with any of the clustering and filtering approaches, although recent studies show that a non‐destructive approach can counteract this bias (Marquina et al. 2019). Furthermore, different degrees of sclerotization among the different taxa may impact the quantity of extracted DNA, particularly when using a non‐destructive approach as in our study (Erdozain et al. 2019; Kirse et al. 2023; Marquina et al. 2019; Zizka et al. 2018). While size‐sorting and a destructive extraction method may potentially increase the amount of extracted DNA (and theoretically the number of identified species), they also present significant drawbacks. These methods do not alleviate the issues related to primer and mass biases, as discussed earlier. Furthermore, they prevent the possibility of re‐checking voucher specimen after metabarcoding—a critical step for validation and verification in biodiversity studies (Remmel et al. 2024). Therefore, the non‐destructive extraction method has the significant benefit of preserving vouchers, enabling subsequent taxonomic analysis. This preservation is particularly crucial in the largely understudied taxa of Diptera and Hymenoptera, often referred to as “Dark Taxa” (Chimeno et al. 2022; Hausmann, Krogmann, et al. 2020). In the current context, despite advancements in DNA metabarcoding, applications of species‐level data in Hymenoptera still heavily rely on results obtained through morphological identification rather than solely on metabarcoding outcomes.

In the particular case of the family Syrphidae, our exemplary family in the present survey, the high dissimilar community between the DNA metabarcoding and morphology is unexpected. Cross‐contamination and shared COI haplotypes can be a source of mismatch between the two approaches, as syrphids are frequent prey of predaceous arthropods such as Diptera and Hymenoptera (Blösch 2000; Gilbert 2005; Pickard 1975) and certain genera have species with shared COI haplotypes (Dietz et al. 2023; Haarto and Ståhls 2014; Locke and Skevington 2013; Young, Marshall, and Skevington 2016). But the COI haplotype is not common among all the syrphid genera and would not explain all the cases of discrepancies, for example, the detection of *Episyrphus balteatus*, *Eristalis tenax*, or *Helophilus pendulus* in the OTU96 from 2016. Should the Syrphidae include taxa or species groups characterized by minimal interspecific differentiation, the aggregation of multiple species within a single OTU/ASV becomes a likely outcome. Still, this would not explain the detection of genera using metabarcoding that were not present among the morphological species such as *Dasysyrphus* or *Episyrphus* in the 2016 sample. The interspecific divergence in the subfamily Syrphinae exhibits significant overlap with the intraspecific divergence distribution, thereby negating the presence of a general DNA barcoding gap for hoverflies as a group (Jordaens et al. 2015; Meier, Zhang, and Ali 2008); this overlap is not as frequent in the subfamily Eristalinae as in Syrphinae, but it exists. *Eristalis tenax* or *H. pendulus* belong to Eristalinae, and although *E. balteatus* is a Syrphinae, it is the single species of the genus occurring in Europe. Thus, the detection of *E. balteatus* in the OTU96 from 2016 implies not only a species not found in the morphological survey but a genus not studied morphologically. We must point out that all the hoverflies identified morphologically, as well as those identified only by metabarcoding, have reference sequences in BOLD; hence, the lack of a reference barcode in the database cannot explain the observed discrepancies.

In addition, although morphological misidentification is likely to occur, Syrphidae is a well‐studied flower‐visitor group with several good identification tools for northern and central Europe (Bartsch 2009a, 2009b; Bot and Van de Meutter 2019, 2023; Speight and Sarthou 2017; Van Veen 2010), and the collected species in 2016 and 2017 (Table S3) do not represent a serious challenge in their morphological identification, with the exception of some females or partially destroyed specimens. Inaccuracies in reference databases are very likely to occur and we cannot rule it out completely, although in a minor percentage for Syrphidae as the community of syrphid researchers helped to build a well‐curated database for GBOL and other parts of the world.

The biomass bias with unique organisms or single species showing lower detection rates (Strutzenberger et al. 2024) could be argued as the absence of certain species identified morphologically in any DNA metabarcoding approach, such as *Triglyphus primus* from 2016 or *Eupeodes luniger* from 2017, but not for the non‐detection of other species with more specimens, that is, *Platycheirus* species other than *P. clypeatus* (Table S3). But this biomass bias cannot explain the relatively high number of species detected by DNA metabarcoding that were not found in our morphological survey, especially as the detected species are mostly medium‐to‐large‐sized syrphids, that is, *E. tenax* , *Dasysyrphus tricinctus*, *H. pendulus*, or *Syrphus ribesii* . Based on our experience, and corroborated by the GBOL database, these species show a barcoding gap with the nearest neighbor species (in other words, the intra‐ and the interspecific *p*‐distance do not overlap) and they are very common and abundant species in Central Europe, which makes the overlooking by us in the morphology survey unlikely.

Our findings mirror other works where morphologically identified species are compared with metabarcoding species results (Remmel et al. 2024; with *J* = 0.5 for Syrphidae). Thus, the high Jaccard dissimilarity index calculated for Syrphidae in the present study might be explained by other reasons, such as the accuracy of the DNA mini‐barcode of 313 bp in length used in metabarcoding to identify species. While DNA mini‐barcodes are commonly used in metabarcoding, there is a concern about whether they might affect the accuracy of identifying syrphid species, especially when compared to full‐length 658 bp COI barcodes, as shorter sequences may not provide enough genetic information for precise species identification, at least for some species. Many studies comparing DNA mini‐barcodes and morphology are based on mock communities (Aylagas et al. 2016; Baloğlu et al. 2021; Govender et al. 2022), which may not fully capture the complexities present in real‐world scenarios. This suggests that the observed mismatch could be influenced by factors unique to natural environments, such as the presence of closely related species and environmental variables affecting DNA extraction and amplification. Srivathsan et al. (2021), for example, found no difference in the number of species between full‐length DNA barcodes (658 bp) and the 313‐bp fragments, although they did not state if the species composition was highly similar or not. Identifying syrphid species based on full‐length DNA barcodes accurately can be challenging due to various factors such as genetic variability within species and incomplete reference databases, as mentioned. This raises the question of whether DNA mini‐barcodes exacerbate this issue or if it is inherent to metabarcoding techniques in general. Without a better explanation, it seems that the detection of these species not present in our morphological study may be due to cross‐contamination.

Despite the current limitations in DNA metabarcoding, we also highlight the potential of a non‐destructive DNA metabarcoding approach for uncovering cryptic diversity in highly diverse groups, which morphologically can still be very challenging. We emphasize the potential of enhancing DNA metabarcoding results not only through improvements in the DNA extraction and PCR processes but also by refining the final assessment through appropriate bioinformatic analysis, particularly focusing on the clustering and filtering approaches. This is especially important in studies where the metabarcoding analysis cannot be cross‐validated with morphology.

## 5 Conclusion

While DNA metabarcoding has become a valuable tool for insect biomonitoring assessments, there are still many limitations that require attention, especially during the in silico stage where the impact of clustering and filtering approaches is significant. Our study highlights that for Brachycera clustering ASVs into OTUs at 97% cutoff and subsequently applying LULU filtering at a 96% minimum match yields the most interesting results. However, for Hymenoptera, the same approach resulted in considerably different estimates compared to diversity assessed via morphological identification. Therefore, we advocate for a species composition analysis whenever possible. Despite the variations in results and resolution observed for Brachycera and Hymenoptera, and the persistent limitations in the application of this methodology, we recognize the potential of achieving high species‐level resolution with a non‐destructive DNA metabarcoding approach. This approach not only retains for future analyses but also preserves the entire sample as a voucher. This preservation is particularly valuable for large‐scale monitoring programs utilizing DNA metabarcoding as a standard methodology. Addressing these limitations and optimizing protocols will enhance the reliability and accuracy of DNA metabarcoding for insect biomonitoring in the future.

## Author Contributions

**Isabel C. Kilian:** conceptualization (equal), data curation (lead), formal analysis (lead), investigation (lead), methodology (equal), validation (equal), visualization (lead), writing – original draft (lead), writing – review and editing (lead). **Ameli Kirse:** conceptualization (equal), data curation (equal), formal analysis (supporting), methodology (equal), validation (equal), writing – original draft (supporting), writing – review and editing (equal). **Ralph S. Peters:** conceptualization (equal), funding acquisition (equal), supervision (equal), validation (equal), writing – original draft (supporting), writing – review and editing (equal). **Sarah J. Bourlat:** conceptualization (supporting), funding acquisition (supporting), supervision (supporting), validation (supporting), writing – review and editing (equal). **Vera G. Fonseca:** conceptualization (equal), funding acquisition (lead), supervision (supporting), validation (supporting), writing – review and editing (equal). **Wolfgang J. Wägele:** funding acquisition (equal), supervision (supporting), writing – review and editing (equal). **Andrée Hamm:** funding acquisition (supporting), project administration (lead), writing – original draft (supporting), writing – review and editing (supporting). **Ximo Mengual:** conceptualization (equal), funding acquisition (equal), supervision (equal), validation (equal), writing – original draft (supporting), writing – review and editing (equal).

## Conflicts of Interest

The authors declare no conflicts of interest.

## Supporting information


Appendix S1

## Data Availability

Sequencing data generated and analyzed in this study are publicly available through the Genbank SRA archive (accession nos. PRJNA1105927). The datasets are publicly available in Mendeley data (https://doi.org/10.17632/pccc22sd4w.2).
