# Supplementary material for: Maximizing Identification Precision of Hymenoptera and Brachycera (Diptera) With a Non‐Destructive DNA Metabarcoding Approach
Source: Ecol Evol. 2025 Jan 23;15(1):e70770. doi: 10.1002/ece3.70770 (PMC11756930; doi:10.1002/ece3.70770)
Supplement: Supplementary file 1 — Appendix S1 [file ECE3-15-e70770-s001.docx]

**Supporting information**

**Table S1**: Publications with identification keys used in the morphological survey of the samples.

| **Taxa** | **Identification keys** |
| --- | --- |
| Hymenoptera | Amiet et al., 2012; Benson, 1951, 1958; Bitsch, 1992; Gokcezade et al., 2010; Goulet et al., 1993; Prous et al., 2019; Schmid-Egger & Scheuchl, 1997, 1997; Stresemann & Klausnitzer, 2011; Witt, 1998 |
| Brachycera | Drake, 1993; Gregor et al., 2016; Naglis, 2012; Oosterbroek, 2006; Rozkosny & Frantisek, 2004; Stresemann & Klausnitzer, 2011; Tschorsnig, 1994; Van Emden, 1954; Zeegers, 1992 |
| Syrphidae | Bartsch, 2009a, 2009b; Haarto & Ståhls, 2014; Van Veen, 2010 |


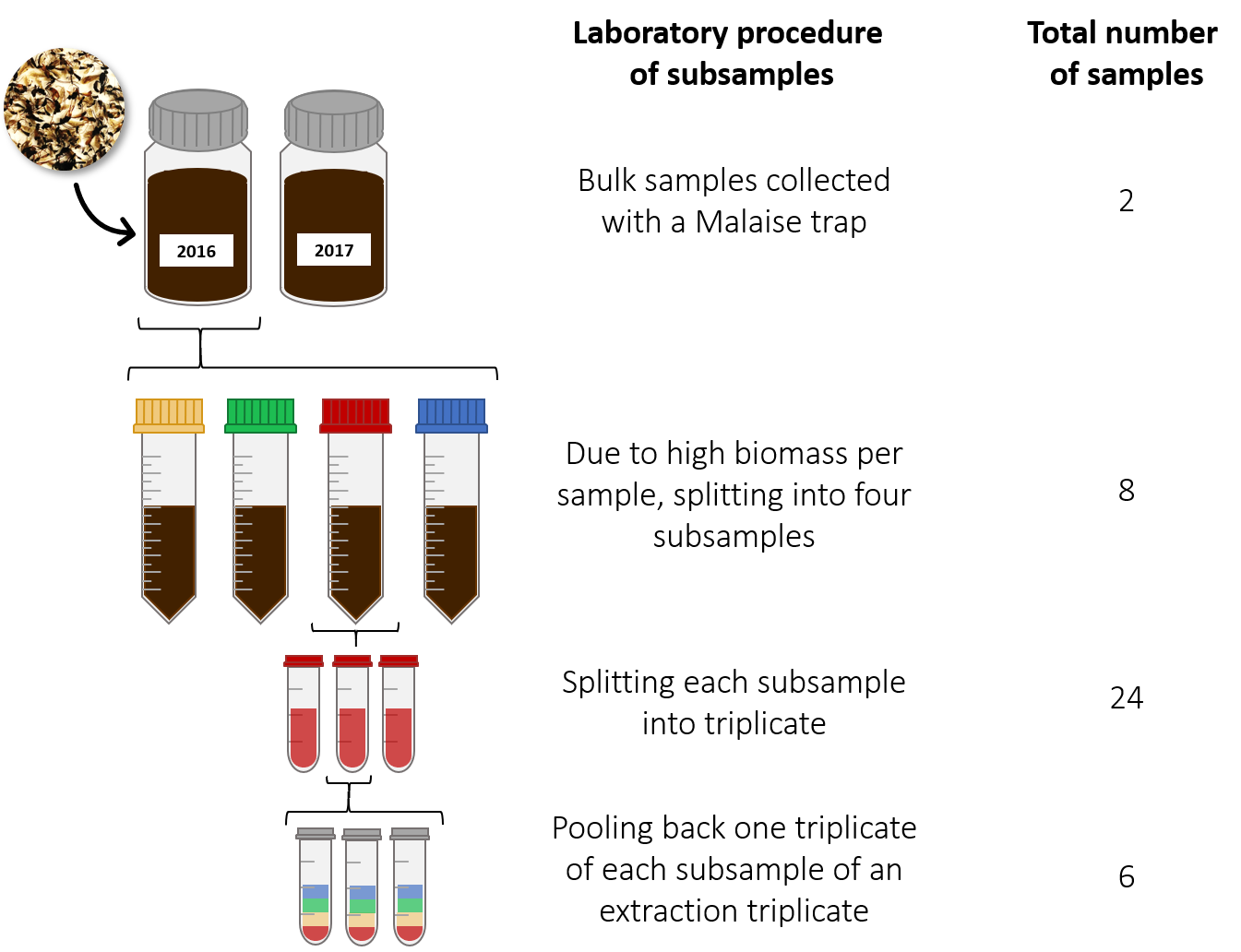


**Figure S1:** Experimental setup for DNA extraction. The specimens of two Malaise trap bulk samples were split into four equal subsamples after drying. Each subsample was mixed with an extraction buffer. After digestion, the lysis solution was split into three replicates per subsample, which were pooled back together to three extraction replicates after the second filtering process. For a more detailed description, see Material and Methods section.

**Table S2**: Final list of Hymenoptera (excluding Ichneumonoidea) and Brachycera diversity identified via DNA-metabarcoding applying four different clustering approaches compared to the morphologically identified diversity: 1) LULU-filtered ASVs at a minimum match of 84% (default settings; ASV84); 2) LULU-filtered ASVs at a minimum match of 96% (ASV96); 3) ASVs clustered to OTUs at 97% similarity cutoff and LULU-filtered at a minimum match of 84% (default settings; OTU84); and 4) ASVs clustered to OTUs at 97% similarity cutoff and LULU-filtered at a minimum match of 96% (OTU96). Blasted molecular units refer to molecular units identified with a name after blasting.

| **Sample** | **Taxa** | **Unit** | **ASV84** | **ASV96** | **OTU84** | **OTU96** |  | **Morphology** |
| --- | --- | --- | --- | --- | --- | --- | --- | --- |
| **2016** | **Brachycera** | Reads | 54,116 | 62,519 | 62,519 | 54,499 |  |  |
|  |  | Family | 11 | 10 | 10 | 24 | Family | 29 |
|  |  | Genera | 13 | 14 | 14 | 66 |  |  |
|  |  | Molecular unit (MU) | 15 | 16 | 16 | 97 |  |  |
|  |  | Blasted MU | 15 | 16 | 16 | 80 | Morphospecies | 71 |
|  | **Hymenoptera** | Reads | 7 | 7 | 7 | 1,162 |  |  |
|  |  | Family | 1 | 1 | 1 | 2 | Family | 17 |
|  |  | Genera | 1 | 1 | 1 | 5 |  |  |
|  |  | Molecular unit (MU) | 1 | 1 | 1 | 13 |  |  |
|  |  | Blasted MU | 1 | 1 | 1 | 11 | Morphospecies | 36 |
|  | **Syrphidae** | Reads | 14,602 | 31,266 | 31,266 | 18,433 |  |  |
|  |  | Genera | 3 | 5 | 5 | 10 | Genera | 6 |
|  |  | Molecular unit (MU) | 3 | 5 | 5 | 11 |  |  |
|  |  | Blasted MU | 3 | 5 | 5 | 11 | Species | 12 |
| **2017** | **Brachycera** | Reads | 51,311 | 47,415 | 47,415 | 46,781 |  |  |
|  |  | Family | 11 | 10 | 10 | 31 | Family | 31 |
|  |  | Genera | 14 | 15 | 15 | 64 |  |  |
|  |  | Molecular unit (MU) | 16 | 18 | 18 | 119 |  |  |
|  |  | Blasted MU | 16 | 17 | 17 | 96 | Morphospecies | 75 |
|  | **Hymenoptera** | Reads | 644 | 746 | 746 | 746 |  |  |
|  |  | Family | 3 | 3 | 3 | 3 | Family | 22 |
|  |  | Genera | 4 | 4 | 4 | 4 |  |  |
|  |  | Molecular unit (MU) | 5 | 5 | 5 | 5 |  |  |
|  |  | Blasted MU | 5 | 5 | 5 | 5 | Morphospecies | 59 |
|  | **Syrphidae** | Reads | 26,827 | 28,449 | 28,449 | 29,075 |  |  |
|  |  | Genera | 3 | 5 | 5 | 11 | Genera | 8 |
|  |  | Molecular unit (MU) | 3 | 5 | 5 | 16 |  |  |
|  |  | Blasted MU | 3 | 5 | 5 | 15 | Species | 11 |
| **Both years combined** | **Brachycera** | Reads | 105,427 | 109,934 | 109,934 | 101,280 |  |  |
|  |  | Family | 16 | 15 | 15 | 34 | Family | 35 |
|  |  | Genera | 20 | 21 | 21 | 95 |  |  |
|  |  | Blasted MU | 23 | 24 | 24 | 144 | Morphospecies | 114 |
|  | **Hymenoptera** | Reads | 651 | 753 | 753 | 1,908 |  |  |
|  |  | Family | 3 | 3 | 3 | 3 | Family | 27 |
|  |  | Genera | 4 | 4 | 4 | 8 |  |  |
|  |  | Blasted MU | 5 | 5 | 5 | 15 | Morphospecies | 85 |
|  | **Syrphidae** | Reads | 41,429 | 59,715 | 59,715 | 47,508 |  |  |
|  |  | Genera | 4 | 6 | 6 | 14 | Genera | 12 |
|  |  | Blasted MU | 6 | 10 | 10 | 26 | Species | 21 |


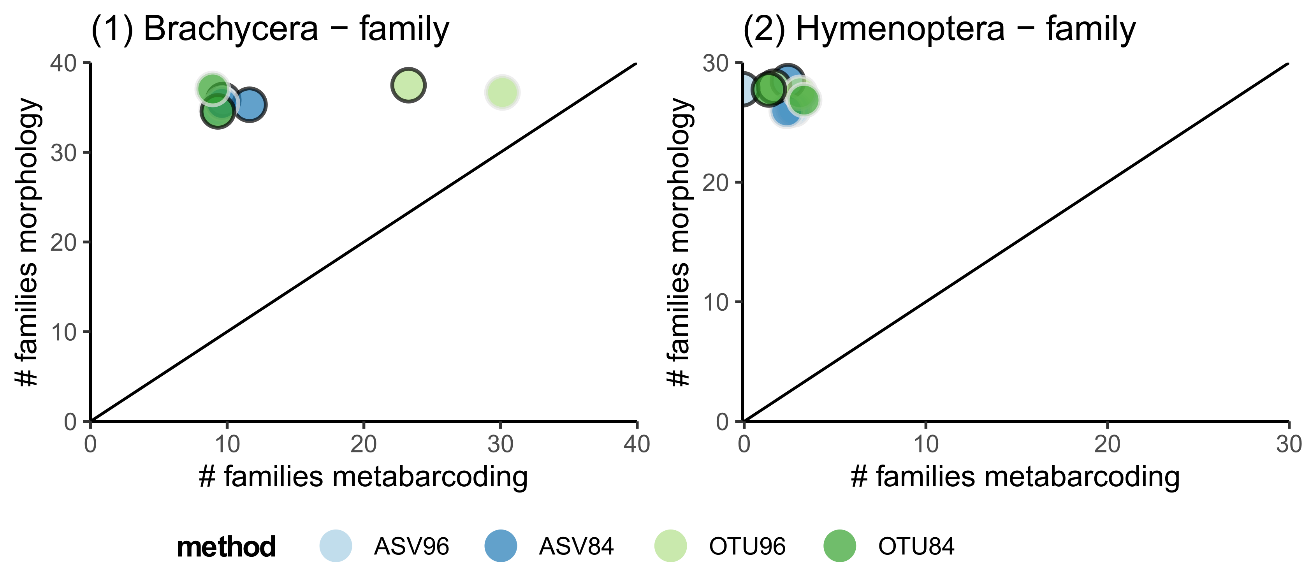


**Figure S2**: Comparison between number of families (1) of Brachycera and (2) of Hymenoptera identified with morphological identification and DNA metabarcoding with four different clustering approaches across both samples of 2016 and 2017. ASVs were either clustered in OTUs at 97% similarity cutoff and LULU-filtered using 1.) standard settings at 84% minimum match (OTU84) or 2.) using a 96% minimum match (OTU96), or ASVs were just directly LULU-filtered using the standard settings at 3.) 84% minimum match (ASV84) or 4.) at 96% minimum match (ASV96). The solid line represents a 1:1 relationship. Points with black borders represent the sample of 2016, and points with grey borders represent the sample of 2017.

**Table S3:** Final list of Syrphidae diversity identified via DNA-metabarcoding applying four different clustering and filtering approaches (qualitative data) and morphological identification (quantitative data). ASV84 = LULU-curated ASVs using standard settings at 84% minimum match, ASV96 = LULU-curated ASV using a 96% minimum match, OTU84 = ASVs clustered in OTUs at 97% similarity cutoff and LULU filtered using the standard settings at 84% minimum match, OTU96 = ASV clustered to OTUs at 97% similarity threshold and LULU filtered using a 96% minimum match.

|  | **2016** | | | | | **2017** | | | | |
| --- | --- | --- | --- | --- | --- | --- | --- | --- | --- | --- |
| **Syrphid species** | ASV84 | ASV96 | OTU84 | OTU96 | Morph.* | ASV84 | ASV96 | OTU84 | OTU96 | Morph.* |
| *Dasysyrphus tricinctus* | 0 | 0 | 0 | 1 | 0 | 0 | 0 | 0 | 0 | 0 |
| *Episyrphus balteatus* | 0 | 0 | 0 | 1 | 0 | 0 | 0 | 1 | 0 | 18 |
| *Eristalinus sepulchralis* | 0 | 0 | 0 | 0 | 0 | 0 | 0 | 1 | 0 | 1 |
| *Eristalis tenax* | 1 | 1 | 1 | 1 | 0 | 1 | 1 | 1 | 1 | 0 |
| *Eristalis arbustorum* | 0 | 0 | 0 | 0 | 0 | 0 | 0 | 1 | 0 | 1 |
| *Eristalis intricaria* | 0 | 0 | 0 | 0 | 0 | 0 | 0 | 1 | 0 | 1 |
| *Eupeodes corollae* | 0 | 0 | 0 | 0 | 0 | 0 | 0 | 1 | 0 | 0 |
| *Eupeodes* sp. | 0 | 0 | 0 | 0 | 0 | 0 | 0 | 1 | 0 | 0 |
| *Eupeodes luniger* | 0 | 0 | 0 | 0 | 0 | 0 | 0 | 0 | 0 | 2 |
| *Helophilus pendulus* | 0 | 1 | 1 | 1 | 0 | 0 | 0 | 0 | 0 | 0 |
| *Helophilus trivittatus* | 0 | 0 | 0 | 0 | 0 | 0 | 0 | 1 | 0 | 1 |
| *Fagisyrphus cinctus* | 0 | 0 | 0 | 1 | 0 | 0 | 0 | 0 | 0 | 0 |
| *Melanostoma mellinum* | 1 | 1 | 1 | 1 | 109 | 1 | 1 | 1 | 1 | 45 |
| *Melanostoma* sp. | 0 | 0 | 0 | 1 | 0 | 0 | 0 | 0 | 0 | 0 |
| *Melanostoma scalare* | 0 | 0 | 0 | 0 | 5 | 0 | 0 | 0 | 0 | 0 |
| *Paragus* sp. | 0 | 0 | 0 | 1 | 1 | 0 | 0 | 1 | 0 | 0 |
| *Platycheirus clypeatus* | 1 | 1 | 1 | 1 | 3 | 0 | 1 | 1 | 1 | 0 |
| *Platycheirus* sp. | 0 | 0 | 0 | 0 | 1 | 0 | 0 | 0 | 0 | 0 |
| *Platycheirus albimanus* | 0 | 0 | 0 | 0 | 3 | 0 | 0 | 0 | 0 | 0 |
| *Platycheirus angustatus* | 0 | 0 | 0 | 0 | 1 | 0 | 0 | 0 | 0 | 0 |
| *Platycheirus europaeus* | 0 | 0 | 0 | 0 | 13 | 0 | 0 | 0 | 0 | 0 |
| *Platycheirus inmaculatus* | 0 | 0 | 0 | 0 | 3 | 0 | 0 | 0 | 0 | 0 |
| *Rhingia campestris* | 0 | 0 | 0 | 1 | 1 | 0 | 0 | 0 | 0 | 0 |
| *Scaeva pyrastri* | 0 | 0 | 0 | 0 | 0 | 0 | 0 | 1 | 0 | 0 |
| *Sphaerophoria scripta* | 0 | 1 | 1 | 1 | 3 | 0 | 1 | 1 | 1 | 15 |
| *Sphaerophoria* sp. | 0 | 0 | 0 | 0 | 0 | 0 | 0 | 0 | 0 | 16 |
| *Sphaerophoria taeniata* | 0 | 0 | 0 | 0 | 0 | 0 | 0 | 0 | 0 | 1 |
| *Syrphus ribesii* | 0 | 0 | 0 | 0 | 0 | 1 | 1 | 1 | 1 | 0 |
| *Syrphus vitripennis* | 0 | 0 | 0 | 0 | 0 | 0 | 0 | 1 | 0 | 2 |
| *Triglyphus primus* | 0 | 0 | 0 | 0 | 1 | 0 | 0 | 0 | 0 | 0 |
| syrphidae molecular unit | 0 | 0 | 0 | 0 | 0 | 0 | 0 | 1 | 0 | 0 |

**References (Supplements)**

Amiet, F., Herrmann, M., Müller, A., & Neumeyer, R. (2012). Fauna Helvetica 26. Apidae 6. Andrena, Melitturga, Panurginus, Panurgus. *entomologische berichten*, *72*, 1–2.

Bartsch, H. (2009a). *Nationalnyckeln till Sveriges flora och fauna. Tvåvingar: Blomflugor: Diptera: Syrphidae: Syrphinae.* ArtDatabanken, SLU, Uppsala.

Bartsch, H.(2009b). *Nationalnyckeln till Sveriges flora och fauna. Tvåvingar: Blomflugor: Diptera: Syrphidae: Eristalinae & Microdontina.* Artdatabanken, Sveriges lantbruksuniversitet, SLU, Uppsala.

Benson, R. B. (1951). Handbook for the identification of British insects. Hymenoptera 2. *Symphyta. Section a. R. Entomol. Soc.(London)*, *2*, 1–49.

Benson, R. B. (1958). Hymenoptera. 2. Symphyta. *Handbooks for the identification of British insects*, *4*, 139–252.

Bitsch, J. (1992). Hermann Dollfuss.—Bestimmungsschlüssel der Grabwespen Nord-und Zentraleuropas (Hymenoptera, Sphecidae), mit speziellen Angaben zur Grabwespenfauna Oesterreichs. 1991; Stapfia, n° 24 (Publikation der botanischen Arbeitsgemeinschaft am O. Oe., Landesmuseum, Museumstrasse 14, A-4010 Linz, Autriche). *Bulletin de la Société entomologique de France*, *97*(1), 32–32.

Drake, C. M. (1993). A review of the British Opomyzidae (Diptera). *British Journal of Entomology and Natural History*, *6*(4), 159–176.

Gokcezade, J. F., Gereben-Krenn, B. A., Neumayer, J., & Krenn, H. W. (2010). *Feldbestimmungsschlüssel für die Hummeln Österreichs, Deutschlands und der Schweiz (Hymenoptera, Apidae)*. Biologiezentrum/Oberösterreichische Landesmuseen.

Goulet, H., Huber, J. T., & Branch, C. A. C. R. (1993). *Hymenoptera of the World: An Identification Guide to Families*. Agriculture Canada.

Gregor, F., Rozkošný, R., Barták, M., & Vaňhara, J. (Hrsg.). (2016). *Manual of Central European Muscidae (Diptera): Morphology, taxonomy, identification and distribution*. Schweizerbart Science Publishers.

Haarto, A., & Ståhls, G. (2014). When mtDNA COI is misleading: Congruent signal of ITS2 molecular marker and morphology for North European *Melanostoma* Schiner, 1860 (Diptera, Syrphidae). *ZooKeys*, *431*, 93–134. https://doi.org/10.3897/zookeys.431.7207

Naglis, S. (2012). Bestimmungsschlüssel für die Gattungen der Langbeinfliegen der Schweiz (Diptera, Dolichopodidae). *Mitteilungen der Schweizerischen Entomologischen Gesellschaft*, *85*(3–4), 251–266.

Oosterbroek, P. (2006). *The European Families of the Diptera: Identification-Diagnosis-Biology*. Brill.

Prous, M., Liston, A., Kramp, K., Savina, H., Vårdal, H., & Taeger, A. (2019). The West Palaearctic genera of Nematinae (Hymenoptera, Tenthredinidae). *ZooKeys*, *875*, 63.

Rozkosny, R., & Frantisek, G. (2004). *Insecta: Diptera: Muscidae. Süßwasserfauna von Mitteleuropa, Bd. 21/29*. Spektrum Akademischer Verlag, Berlin.

Schmid-Egger, C., & Scheuchl, E. (1997). *Illustrierte Bestimmungstabellen der Wildbienen Deutschlands und Österreichs: Andrenidae*. Eigenverl. des Verf.

Stresemann, E., & Klausnitzer, B. (2011). *Stresemann—Exkursionsfauna von Deutschland, Band 2: Wirbellose: Insekten*. Spektrum Akademischer Verlag.

Tschorsnig, H.-P. (1994). Die Raupenfliegen (Diptera: Tachinidae) Mitteleuropas: Bestimmungstabellen und Angaben zur Verbreitung und Okologie der einzelnen Arten. *Stuttgarter Beitrage zur Naturkunde (A)*, *506*, 1–170.

Van Emden, F. I. (1954). Diptera: Cyclorrhapha Calyptrata (I), Section (a). Tachinidae and Calliphoridae. *Handbooks for the identification of British insects*, *10*(4), 1–133.

Van Veen, M. P. (2010). *Hoverflies of Northwest Europe: Identification Keys to the Syrphidae*. KNNV Publishing.

Witt, R. (1998). *Wespen: Beobachten, bestimmen*. Naturbuch-Verlag. https://books.google.de/books?id=XeZMAAAAYAAJ

Zeegers, T. W. P. (1992). *Tabel voor de grotere sluipvliegen en horzels van Nederland*. Jeugdbondsuitgeverij.
